# Effectiveness evaluation of a health promotion programme in primary schools: a cluster randomised controlled trial

**DOI:** 10.1186/s12889-016-3330-4

**Published:** 2016-07-30

**Authors:** Ludwig Grillich, Christina Kien, Yanagida Takuya, Michael Weber, Gerald Gartlehner

**Affiliations:** 1 Department for Evidence-based Medicine and Clinical Epidemiology, Danube University Krems, Dr. Karl Dorrek Straße 30, 3500 Krems an der Donau, Austria; 2 School of Applied Health and Social Sciences, University of Applied Sciences Upper Austria, Wels, Austria; 3 Medical University of Vienna, Wien, Austria; 4 RTI International, Durham, USA

**Keywords:** Child health, Public health, Health promotion, Schools, Interventions, Effectiveness evaluation, RCT

## Abstract

**Background:**

Programmes based on the World Health Organization’s Health Promoting Schools framework (HPS) have been implemented in several countries but for evidence-based policy-making more research is required to determine the effectiveness of the HPS approach.

**Methods:**

We conducted a cluster randomised controlled trial. The units of randomisation were primary school classes recruited in May 2010. Eligible participants were Year 3 primary school classes in Lower Austria that had not participated in a similar programme during the last two years. After baseline assessment in September 2010, 53 classes from 45 primary schools in Lower Austria were randomly assigned to an intervention (*n* = 26 classes, 432 children) or waiting control arm (*n* = 27 classes, 493 children aged 8.7 years +/- 4 months). Over the course of 1.5 academic years, participating teachers received on-the-job training (20 h) and two workshops (8 h) to promote health related behaviour in students such as physical activity during the school day and to improve the quality of regular physical education classes. We assessed 15 outcomes grouped into five categories: Emotional and Social Experience in School, Physical Activity, Well-being, and Attention Performance measured by validated and standardised questionnaire and Motor Skills measured by validated and standardised motoric and coordination tests in the school gym. The primary outcome was Classroom Climate and part of the outcomecategory Emotional and Social Experience in School. The final assessment took place in April 2012. All assessors were blinded to the allocation of classes. Multilevel growth modelling was used to investigate programme effectiveness.

**Results:**

We could not detect any statistically significant differences between groups for the outcomecategories Emotional and Social Experience in school (*p* = 0.22 to 0.78), *Physical Activity*, *Well-being*, and *Attention Performance*. Significant differences between groups were limited to the outcomecategory *Motor Skills* (Complex Reaction Ability, Spatial Orientation Skills, Coordination with Precision) which were higher in the intervention group (*P* < .05).

**Conclusions:**

Despite small statistically significant differences in Motor Skills, our study could not detect any clinically relevant improvements in the Emotional and Social Experience at School (including the primary outcome ClassroomClimate), Physical Activity, Well-being, Motor Skills and Attention Performance of students.

**Trial registration:**

German register of clinical studies: DRKS00000622. Retrospectively registered: 03.12.2010. Approved by the Ethics Committee of Lower Austria (GS4-EK-4/107-2010).

## Background

Physical activity is an important determinant of healthy physical and psychological development in children [1]. It improves the development of motor skills, [2] enhances self-esteem, [3] leads to better psychological well-being, and strengthens cardiorespiratory fitness [4]. Despite the clear health benefits of physical activity, evidence [5] suggests that a significant proportion of children and adolescents do not meet World Health Organization (WHO) recommendations for daily physical activity: for children and adolescents aged 5-17 years at least 60 min of physical activity daily in moderate to vigorous intensity (MVPA) [6, 7]. International surveys of physical activity highlight that fewer than 50 % of boys and girls meet the WHO standards and are active enough to achieve health benefits [7–11] from physical exercise where males show higher frequency of daily MVPA than females [12] and low family affluence corresponds with lower frequency of daily MVPA [13]. For example, in Australia only 22 % of boys and 20 % of girls age 9–11 years meet the WHO standards [14], whereas in the United States, 49 % of boys and 35 % of girls accumulated at least 60 min of MVPA [12]. In Austria 34.8 % children aged 11 years meet the WHO standards and this rate drops to 8.7 % for children aged 17 years [15, 16]. This age-related declines in MVPA are commonly reported in literature [17, 18] and starts in primary school [19, 20].

This situation is alarming for two main reasons: first, physical activity habits established during childhood and adolescence are likely to be carried through into adulthood [21, 22]. Second, in adults, physical inactivity and lack of fitness are associated with increased cardiovascular risk factors [23] independent of body weight [24]. Consequently, the decrease in physical activity over the past decades is one of the main causes of the increase in obesity, [25] a serious public health issue worldwide [26]. According to self-reported height and weight in Austria, 11 % of girls and 15 % of boys are overweight or obese. Associated health-related problems include sleep apnoea and orthopaedic problems; psychosocial repercussions, such as poor self-image; stigmatization and depression; and impaired quality of life [13, 16]. Modelling studies show that a 10 % increase in physical activity in a population could result in significant savings in health care costs [9, 27, 28]. Therefore, physical activity was already labelled as “today’s best buy in public health” two decades ago [29].

There are several reasons for lack of physical activity and it cannot be attributed solely to personal characteristics. So countries that are tackling this complex issue are increasingly electing to employ multi-component approaches (that is informational, behavioural, and environmental) in increasing a population’s physical activity [30–32]). The authors of a recent Cochrane review about community-wide interventions for increasing physical activity [33] found a noticeable inconsistency in the findings in the available studies. They draw the conclusion that the body of evidence does not support the hypothesis that the multi-component community-wide interventions studied effectively increased physical activity for the population. The absence of an effect could be explained by a failure to reach the whole community, especially lower socio-economic groups. If health promotion programs do not reach lower socio-economic groups as they do high socio-economic groups, the result could be a widening of the gap in health inequality [34]. In contrast to this risk of broad community-wide interventions, school-based interventions reach children of different socio-economic backgrounds as well as those who might have dropped out if given a free choice. Schools are an important setting for interventions to promote health in children. In developed countries children spend a substantial part of their time in schools and school-based interventions give all children an equal chance to benefit.

In a systematic review, Barr-Anderson et al. described 14 different school-based interventions for physical activity in primary schools [35]. Most of these interventions were implemented as additional interventions, for example additional short physical activity breaks or physical activity homework. Another approach to promote health in schools is based on the World Health Organization’s Health Promoting Schools (HPS) framework [36]. HPS can be regarded as another way of schooling rather than an add-on programme [37]. Although definitions vary, the characteristics of a HPS are the school curriculum (health topics are promoted through the formal school curriculum), ethos (health related values and attitudes - like the value of physical activity - guiding the daily teaching routine in the school) and involvement of families and outside agencies to promote health and well-being. The HPS framework integrates health-related behaviour - like physical activity - into the regular academic curriculum (termed “integrated approach”). The goal is to modify the daily teaching routine towards creating a positive and healthy learning environment. In such an approach pupils have the opportunity for physical activity during regular class time. In “running dictation”, for example, the class is divided into teams. In each team there is a reader and a writer while a short text is located at the back of the classroom. The reader has to run to the text to read and memorize a part of it. As quickly as possible the reader will run back and dictate the text to the writer in their group. The writer writes while the reader goes back to read more text. Comparing this with a classical “dictation” where a teacher stands in front of the class dictating text “running dictation” provides many more possibilities for MVPA.

The main benefit of the integrated approach is that no additional lessons or curricula changes are necessary. However, the integrated approach requires training of teachers to enable them to create a healthy and positive learning environment.

To date, the effectiveness of the HPS approach has not yet been fully proven. A Cochrane review assessing the effectiveness of the HPS framework in improving the health and well-being of children [36] calls for more well-designed research to establish its effectiveness. The authors of the Cochrane review found evidence of effectiveness to suggest the HPS approach can produce improvements in certain areas of health, but the quality of evidence was only rated low to moderate and there was no evidence of an effect on mental health and well-being. Therefore, monitoring of the impact of an HPS intervention is needed and impact evaluation is recommended if HPS interventions are implemented.

The objective of our study was to assess the effectiveness of an integrated health promotional programme in Lower Austrian elementary schools based on the HPS framework to increase children’s emotional and social experience, physical activity and well-being at school.

Our hypothesis was that children who are part of an intervention based on the HPS framework made better emotional and social experiences at school, are more physically active, have a higher level of well-being, and develop better motor-skills and attention performance than children in regular classes.

## Methods

The study took place in Lower Austria, the largest province of Austria with a population of 1.612 million inhabitants. The programme “Classes in Motion” and this study were funded by the Lower Austrian Health and Social Fund (trial registration DRKS00000622 – German register of clinical studies) and approved by the Ethics Committee of Lower Austria (GS4-EK-4/107-2010).

### Study population and design

All 5416 teachers with a Year 3 primary school class (students aged 8 to 9 years) in Lower Austria were invited to participate in May 2010. The Lower Austrian Health and Social Fund (NÖGUS) announced the study by written invitation to all 636 schools and on their homepage. Interested teachers registered their class for participation via an internet portal. 558 teachers registered their classes between May and June 2010 (Table 1). Eligible participants (clusters) were Year 3 primary school classes (students aged 8 to 9 years) in Lower Austria that had not participated in a similar programme during the last 2 years. 81 classes fulfilled the eligibility criteria. We sent a written invitation letter to 67 randomly pre-selected classes of which 14 denied participation. The study comprised all pupils in the selected classes for whom their parents had provided informed consent.

**Table 1:** Outcome-groups, outcomes and measuring instruments

Type of outcome-group	Outcomes to be measured	Measuring instrument
Emotional and Social Experience at School	Classroom Climate	Questionnaire on Social and Emotional Experiences at School of Elementary School Children (FEESS 3-4 [40])
	Attitude towards School	
	Feeling Accepted by the Teacher	
Physical Activity	Physical Activity during School Breaks	The Physical Activity Questionnaire for Older Children (PAQ-C) [41]
	Physical Activity Enjoyment	Physical Activity Enjoyment Scale (PACES) [41, 44]
Well-being	Psychological Well-being	KIDSCREEN-52 health-related questionnaire [45]
	Physical Well-being	
	Moods and Emotions	
	Sense of Coherence	Bettge, S. [46]
Motor Skills	Coordination with Precision	subtests from the German Motoric Test [47]
	Coordination under Time Pressure	
	Spatial Orientation Skills	Children’s Coordination Test (KiKo) [48]
	Complex Reaction Ability	
	Kinesthetic Differentiation Ability	
Attention performance	Attention and Concentration	d2 Test [49]

The design of the study was a cluster randomised controlled trial (CRCT) with a follow-up period of 2 years. After baseline assessment 53 school classes from 45 primary schools were randomly assigned to intervention (*n* = 26) or waiting list (*n* = 27) following a computer-generated sequence created by an external statistician. The units of randomisation were school classes, which in the Austrian primary school system are taught mainly by a single teacher. In eight cases two classes from the same school participated in the trial. To prevent carry-over effects from an intervention class to a control class during the trial, classes within the same school were randomised as a single unit to either intervention or waiting list. In the academic years 2010/11 to 2011/12, the programme’s intervention was carried out in the intervention group whereas the control group (waiting list) followed the regular curriculum (without intervention). In the academic year 2012/2013, the control group started with the intervention. The final assessment took place in April 2012.

### Intervention

The intervention Classes in Motion (Bewegte Klasse) based on the HPS framework was funded and implemented by “Healthy Lower Austria” [38]. Training was provided to classroom teachers in primary schools in Lower Austria in collaboration with the Teacher’s College. Over the course of 1.5 academic years, teachers received 20 h of tailored on-the-job training (by a qualified health promotion specialist), partly during the regular classes, covering topics such as active teaching, motivational techniques and safety procedures. Furthermore, teachers participated in two workshops (8 h) which focused on the effects of physical activity, learning theories and practical didactical techniques. The goals of the training were to develop teacher competency, to create a healthy and positive learning environment and to improve the quality of physical education classes. At the beginning of the programme, a qualified health promotion specialist assessed the strengths, weaknesses and the learning needs of teachers to create a healthy and positive learning environment. The teachers in the control group did not receive this intervention.

### Outcome measures

Based on the goals of the intervention, the primary stakeholders (funder and project team) selected 15 outcomes in a participatory process guided by the research team.

The research team grouped the 15 outcomes into five categories: Emotional and Social Experience in School, Physical Activity, Well-being, Motor Skills, and Attention Performance. The primary outcome was Classroom Climate, which was part of the category Emotional and Social Experience in School. Data on outcome measures were collected at baseline in September 2010 (*n* = 51 classes, 925 children) and 20 month follow-up after baseline (*n* = 51 classes, 816 children) in schools using questionnaires and motor coordination tests. All measurements took place at school for all children with trained and blinded assessors within the same four week period. Whereas teacher, children and intervention providers were aware of the allocated arm at follow-up assessment, outcome assessors and data analysts were kept blinded to the allocation. There were no changes to trial outcomes after the trial commenced.

The Swiss Model for Outcome Classification [39] assisted in formulating a logic model and assessing clear objectives and outcome indicators. On the level of determinants of health the model classifies three separate levels: Health-promoting Physical Environment, Health-promoting Social Environment and Health-promoting Individual Resources and Behavioural Patterns. According to the aim of the intervention the primary stakeholder (funder and project manager) expected changes on the levels of Health-promoting Social Environment and Health-promoting Individual Resources and Behavioural Patterns.

At the level of Health-promoting Social Environment the primary stakeholder prioritised Emotional and Social Experience at School (Classroom Climate, A Positive Attitude towards School, Feeling Accepted by the Teacher) as important outcome. At the level of Individual Resources and Behavioural Patterns they prioritised Physical Activity (Physical Activity during School Breaks, Physical Activity Enjoyment), Well-being (Psychological Well-being, Physical Well-being, Moods and Emotions, Sense of Coherence) and Motor Skills (Coordination with Precision, Coordination under Time Pressure, Spatial Orientation Skills, Complex Reaction Ability, Kinaesthetic Differentiation Ability). Classroom Climate was the only outcome at the cluster level and therefore chosen as primary outcome.

#### Emotional and social experience at school

Outcomes included as part of this type of outcome-group were measured by the validated and standardised Questionnaire on Social and Emotional Experiences at School of Elementary School Children (FEESS 3-4 [40]) developed for children in the third and fourth school years. The items of the FEESS ask about children’s attitudes toward emotional and social experiences in school. For each domain, children were asked to rate the truth of several statements (Children indicated on a 4-point- Likert type scale if a statement is exactly true, pretty true, barely true or not true; higher scores indicating better results). The following three subscales were used in this study: Classroom Climate as primary outcomes at cluster level (the extent to which the pupils of a class find themselves sympathetic and do not exclude others because of weakness) which consists of 11 statements; A positive Attitude towards School (the extent a child feels comfortable in school overall) which consists of 14 statements; and Feeling Accepted by the Teacher (the extent a child feels accepted and understood by their teachers) which consists of 13 statements. The testing took place during a regular school lesson in the presence of the teacher and an assessor.

#### Physical activity

Outcomes being part of this outcome-group were Physical Activity during School Breaks and Physical Activity Enjoyment. Physical Activity during School Breaks was measured using The Physical Activity Questionnaire for Older Children (PAQ-C) [41]. The PAQ-C is a self-administered, 7-day recall instrument. It was developed to assess general levels of physical activity throughout the primary school year for pupils in Years 4 to 8 (approximately ages 8-14). The PAQ-C has been supported as a valid and reliable measure of general physical activity levels from childhood to adolescence [42, 43]. The PAQ-C does not discriminate between specific activity intensities, such as moderate and vigorous activities; it simply provides a summary activity score. We used this questionnaire to assess Physical Activity during School Breaks to describe changes in the child’s general level of physical activity. We also used the Physical Activity Enjoyment Scale (PACES) [41, 44] to assess the experience of exercising in pleasant versus unpleasant conditions and between modes of physical activity selected by participants versus modes selected by an investigator. The PACES consists of statements that begin with the stem “When I am physically active…” following pleasant and unpleasant experiences. Children indicated on a 4-point Likert type scale if a statement is exactly true, pretty true, barely true or not true; higher scores indicating better results. The testing took place during a regular school lesson in the presence of the teacher and an assessor.

#### Well-being

Outcomes included as part of this outcome-group were Physical Well-being, Psychological Well-being, Moods and Emotions and Sense of Coherence. Psychological Well-being, Physical Well-being and Moods and Emotions, were measured by the validated and standardised KIDSCREEN-52 health-related questionnaire developed as a self-report measure applicable to healthy and chronically ill children and adolescents aged from 8 to 18 years [45]. The questionnaire consists of items to be answered by the child on a 5-point Likert type scale assessing frequency (never, seldom, quite often, very often, always) or intensity (not at all, slightly, moderately, very, extremely). The research team selected three subscales at the child level: Psychological Well-being (6 items) which includes positive emotions as well as life satisfaction (high values represent a high level of satisfaction and joy while low values represent dissatisfaction and unhappiness) PhysicalWell-being (5 items) which includes level of physical activity, energy and fitness (high values represent active and energetic and low values represent exhausted.) Moods and Emotions (7 items) which shows how familiar a child is with experiences of e.g. loneliness, sadness, and resignation (a high score represents positive mood and feelings of happiness and a low score for depression or bad mood). Additionally, we measured Sense of Coherence (to which extent there is a constant feeling that the challenges of life are structured, predictable and explainable) from Bettge, S. [46]. The testing took place during a regular school lesson the presence of the teacher and an assessor.

#### Motor skills

Outcomes included as part of this outcome-group were Coordination with Precision, Coordination under Time Pressure, Spatial Orientation Skills, Complex Reaction Ability, Kinaesthetic Differentiation Ability. Trained physiotherapists measured these Indicators in the school gym using subtests from the German Motoric Test [47] and the Children’s Coordination Test (KiKo) [48]. The tests lasted approximately one hour. There was a warm up at the beginning (two laps run in circles) and every subtest had a warm-up phase in the form of sample exercises. From the German Motoric Test [47] we used the following two subtests. First, Coordination with Precision which measures coordination ability when the child does precision tasks. The test counts the number of steps a child can balance backwards on a beam without touching the ground.). Secondly, Coordination under Time Pressure. The task for every child was to jump left and right across a highlighted midline with both feet at the same time as quickly as possible. The test counts the number of jumps the child makes in 30 s with high scores indicating good coordination under time pressure. From the Children’s Coordination Test (KiKo) [48] we used three subtests. The first was Spatial Orientation Skills. In this test the child stands 50 cm in front of four numbered medicineballs arranged in a square. Four cards are placed face down lying in the middle of each of the four sides of the square. The task of the child was to run to a side of the square to turn over a card and run to the medicineball written on the card, touch it, run back and then turn over another card. The test counts the number of correct touches in 20 s, high scores indicating better spatial orientation skills. In the subtest Complex Reaction Ability two beams are fixed with one end on a wall bar so that a ball can roll down between the bars. The child stands with his back to the wall bar beside the lower end of the beam. With a start signal a ball rolls down the bars which the child has to stop with both hands. The test counts the distance the ball rolls before the child stops it (low scores indicating better complex reaction ability). In the subtest Kinesthetic Differentiation Ability the child stands on a gymnastics box (80 cm high) and jumps attempting to land with his heel on a line on a gym mat 80 cm in front of the box. The test measures the distance between heels and line (low scores indicating better kinesthetic differentiation ability).

#### Attention performance

Was measured using the validated and standardized d2 Test [49], a cancellation test of attention and concentration. The testing took place during a regular school lesson in the presence of the teacher and an assessor.

To control for potential confounders we also measured sex, age, socioeconomic status (using the Family Affluence Scale [50]) and area of living of children as well as the number of pupils in the classes. To capture fidelity and adverse events 51 teachers who participated filled out a questionnaire at the end of the programme in April 2012.

### Sample size

Primary sample size calculation was based on the primary outcome and an unpaired Student’s *t*-test assuming an effect size of δ = 0.4 (alpha = 0.05, two- sided). Using N Query Advisor 7.0 (Copyright © 1995 – 2007 Janet D. Elashoff) revealed that 100 individuals per group are needed to obtain a power of 80 %. In order to take clustering into account sample size was corrected for the design effect [51] to *n* = 380 per group (average cluster size =20; ICC = 0.2).

### Statistical analysis

Multilevel growth modelling [51, 52] was used to investigate programme effectiveness while taking the nested data structure into account. The R environment [53] using nlme package [54] was used to analyse multilevel models based on three analytic levels (level 1: measurement occasion, level 2: individual, level 3: class). These models adequately consider the nested data structure (i.e., measurement occasion nested in individuals and individuals nested in classes) accounting for the dependencies between observations. Moreover, multilevel models are especially suited for longitudinal data because of their flexibility in modelling the variance-covariance matrix of the residuals [55].

On the measurement level, the model included the predictor time (0 = pretest, 1 = posttest). On the individual level, several covariates were included. At the classroom level, the predictor group (0 = control group, 1 = intervention group) representing the exposure to the intervention was included as predictor. In order to test programme effectiveness, cross-level interaction term time x group representing the differential linear trend of the dependent variable in the intervention group compared to the control group was included. All metric covariates were centred at the grand mean for estimation and interpretation purpose [56].

A series of models were specified to sequentially test the hypotheses. First, we specified a Null Model (Model 0) to compute the intra-class correlation on the individual and class level, which represents the proportion of the variance of the dependent variables at the individual and class level, respectively. Next, we included the predictors intervention and group including cross-level interaction time x group to test for programme effectiveness (Model 1, Hypothesis 1). In the third step, we included covariates to test for programme effectiveness controlling for covariates (Model 2, Hypothesis 2).

The primary analysis was intention-to-treat and involved all pupils who were randomly assigned. Full information maximum likelihood (FIML) estimation under the missing at random (MAR) assumption [57] was used to deal with missing data. That is, the estimation routine uses all available data without discarding incomplete observations [58]. Significance level of all analyses was chosen at α = .05.

## Results

**Fig. 1:**
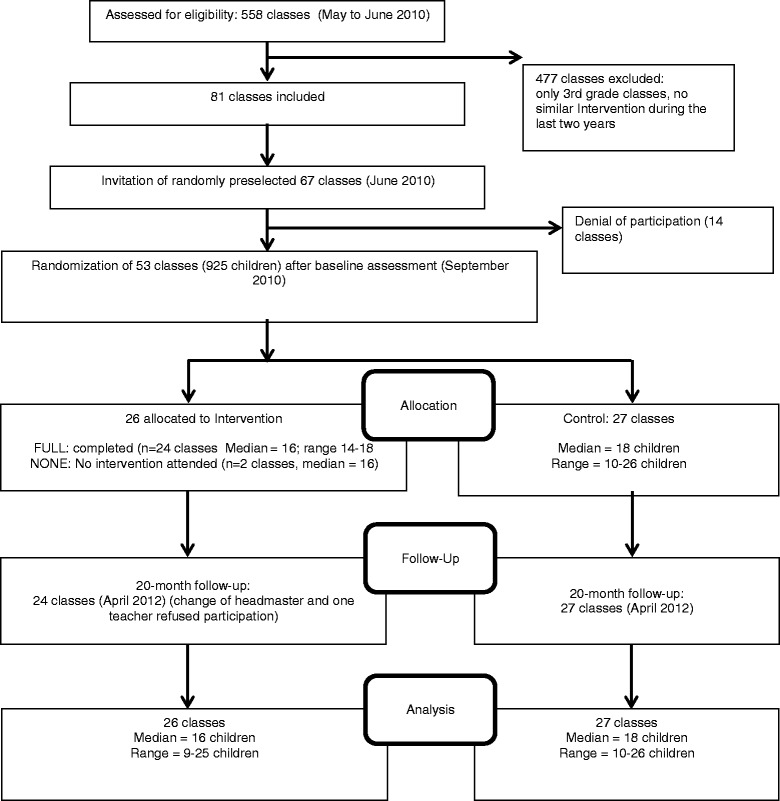
Flow of school classes through study (study cluster)

Figure 1 shows the flow of school classes through the study. 558 teachers registered their classes. 81 matched the eligibility criteria mentioned above. Due to available resources the intervention was designed for a maximum of 60 classes. To compensate for refusals an external statistician randomly selected 67 out of 81 classes for participation. We sent a written invitation letter to the primary teacher of these 67 classes, informing them that they had been accepted but with a 50 % chance to start in autumn 2010 or in autumn 2012. Out of 67 teachers, 53 accepted their classes being part of the study. According to the primary sample size calculation and due to practical reasons (time frame and money) we stopped the acquisition process. Parents provided written consent for 833 children (n_intervention_ = 399; n_control_ = 434) for the psychometric tests and 840 children (n_intervention_ = 397; n_control_ = 443) for the motoric tests. After randomization, two classes in the intervention group refused their commitment to take part in the study. Table 2 shows the baseline characteristics of intervention and control and gives sample size information throughout the trial.

**Table 2:** Baseline characteristics of schoolchildren

Study characteristics, total study population	Intervention group (26 classes)	Control group (27 classes)
Girls, n (%) [N]	189 (48) [391]	218 (53) [409]
Age (years), mean (SD) [N]	8.72 (0.43) [391]	8.71 (0.42) [370]
BMI, mean (SD) [N]	17.95 (3.50) [391]	17.77 (3.00) [419]
Socio-economic level, n (%)[N]		
low	34 (9) [384]	45 (11) [403]
middle	295 (77) [384]	288 (72) [403]
high	55 (14) [384]	70 (17) [403]
Residence, n (%)[N]		
House with garden	323 (84) [386]	314 (78) [403]
Flat with garden	40 (10) [386]	57 (14) [403]
Flat without garden	23 (6) [386]	32 (8) [403]
Hrs/week exercise [N]	3.96 [324]	3.98 [328]

Eight hundred sixteen children completed the baseline and follow-up assessments (12 % loss to follow-up).

Table 3 presents the results. We could not detect a statistically significant difference in the primary outcome Classroom Climate between intervention and control groups (*p* =0.78). Furthermore, except for three outcomes measuring Motor Skills, none of the secondary outcomes were statistically significantly different between treatment groups.

**Table 3:** Correlation between intervention and outcomes (Multilevel growth model)^a^

Type of outcome-group and outcomes	*β* ^b^	ICC class^§^	95 % CI^c^		*p* Value	Score range
			Lower	Upper		
Emotional and Social Experience at School						
Classroom Climate (primary outcome)	-0.02	0.451	-0.16	0.12	0.78	0-3
Attitude towards School	-0.10	0.115	-0.26	0.06	0.22	0-3
Feeling Accepted by the Teacher	-0.03	0.093	-0.13	0.07	0.55	0-3
Physical activity						
Physical activity during School Breaks	0.20	0.116	-0.09	0.49	0.2	-3 - +3
Enjoyment of Physical Activity	0.02	0.014	-0.08	0.12	0.66	1 – 4
Well-being						
Psychological Quality of Life	0.27	0.033	-1.62	2.16	0.78	20.7 – 73.2
Physical Quality of Life	1.11	0.016	-0.76	2.98	0.24	25.2 – 68.5
Moods and Emotions	0.66	0.023	-1.74	3.06	0.59	16.5 – 70.2
Protective Factor “Sense of Coherence”	0.04	0.005	-0.16	0.24	0.66	1 – 5
Motor Skills						
Coordination with Precision	2.58	0.085	0.77	4.39	0.01	0 – 48
Coordination under Time Pressure	0.69	0.081	-0.47	1.85	0.24	5.5 – 62.0
Spatial Orientation Skills	0.46	0.044	0.18	0.74	<.01	0 – 10
Complex Reaction Ability	-12.38	0.173	-17.72	-7.04	<.01	229.5 – 72.5
Kinesthetic Differentiation Ability	-0.32	0.032	-1.18	0.54	0.47	1.13 – 25.83
Attention Performance	1.57	0.070	-2.12	5.26	0.4	6 – 135

^a^ Collective result of multilevel growth model; all covariates were included in the final statistical model (Controlled for sex, age, socioeconomic status, the number of children in the classes)

^b^ Unstandardised regression coefficient

^c^ Confidence interval

§ intraclass correlation coefficient

These three outcomes showed statistically significantly better results for the domain Motor Skills in children of the intervention group. Children in the intervention arm compared with those in the control group had better results for Coordination with Precision (additional 2.58 steps balancing backwards on a beam, *p* =0.01),) Spatial Orientation Skills” (additional 0.46 points in correctly touched balls, *p* < 0.01. Higher scores indicate better spatial orientation skills) and a faster Complex Reaction Ability (12.38 cm less distance till a ball could be stopped, *p* < 0.01. Shorter distance indicates better complex reaction ability). No adverse events were reported.

## Discussion

Our study could not detect any relevant intervention effects of an integrated programme intended to increase Emotional and Social Experience at School (including Classroom Climate as primary outcome), Physical Activity, Motor Skills, Well-being and Attention Performance in primary school children. Three out of five indicators of the outcome category Motor Skills showed statistically significant effects in the expected direction. Any of these effects is small and probably not relevant at the individual level. Bearing in mind that motor skill proficiency levels among young children have the tendency to track into childhood and adolescence [59, 60] and are related to health outcomes such as adiposity [61], and physical activity [62, 63] even small effects at the individual level could have the potential to produce a positive public health effect at the population level.

Considering the design of our study, it was a demanding “real life” intervention trial with strengths and limitations. In interpreting the negative findings, it is important to distinguish between good evidence of ineffectiveness and failure to demonstrate underlying effectiveness [64]. Has the effectiveness evaluation failed to find an effect where one exists, or is there truly no relevant effect of the program “Classes in Movement”?

In the case of our study, confidence intervals were narrow and we are confident that the intervention did not produce any meaningful effects at the individual and class room level. Furthermore, our study included teachers who registered their classes voluntarily to participate in the programme. Even with such highly-motivated teachers, the intervention showed only small effects. Nevertheless, a statistical power of 80 %, as was the case in our study, still means that there is a 20 % likelihood of a false negative result with respect to the primary outcome. Our findings are consistent with other trials which determined the effectiveness of programmes based on the HPS framework that promotes the emotional well-being of students at school [36]. There are only a few studies which were explicitly based upon the HPS framework and collected data on measures of school climate, satisfaction with school and/or well-being. Examples are the study “beyondblue” [65] which sought to reduce depressive symptoms and increase individual-level protective factors and the Gatehouse Project [66] which sought to increase emotional well-being and reduce rates of substance use, known to be related to emotional well-being. Both were three year interventions targeting 13- to 14-year-olds and were implemented in Australia. The intervention “beyondblue” showed no effect for student rating of school climate [65]. The results of the Gatehouse Project showed no effect on unadjusted ORs on low school attachment. However, at final follow up the adjusted ORs suggest an improvement in school attachment among intervention students [66].

There could be several reasons for the lack of observed effects for the intervention in our study. One could be targeting only classes and not the whole school. Looking at Physical Activity in School Breaks for example, it is evident that other children and teacher of the school not being part of the intervention have an influence on this Outcome. Another reason could be the dose of the intervention. Over the course of 1.5 academic years, teachers received 20 h of tailored on-the-job training (by a qualified health promotion specialist). Perhaps that is not enough to show an effect.

Despite the cluster-randomized design, our study has several limitations.

First, to ensure the relevance and adequacy of the outcomes, we conducted a participatory approach to select outcomes that mattered most to the stakeholders of the project. Although we used validated and standardized instruments for children, it is conceivable that these instruments were not sensitive enough to capture relevant effects in Year 3 and Year 4 school children within the follow-up period of our study. Looking at trials that determined the effectiveness of programmes based on the HPS framework that promotes physical activity, a Cochrane review [36] found positive effects for physical activity. The lack in observed effects of the intervention on Physical Activity could also be explained due to the choice of the instrument rather than the intervention. The PAQ-C we used to measure physical activity simply provides a summary activity score which simply might not be sensitive enough to detect effects.

Second, to reduce post-randomisation selection bias, [67] we established a waiting list and all teachers and classes had the opportunity to ultimately implement the programme. Nevertheless, 2 classes (4 % on a cluster level) dropped out. Although a high risk of post-randomisation selection bias is unlikely, it remains unclear how the drop outs influenced results.

Third, due to the characteristics of the intervention, blinding of teachers and pupils was not possible, but assessors were blinded at baseline and followup. It is conceivable though, that a Hawthorne effect (i.e., a change in behaviour and perception of participants because they were part of a study) could have diluted a treatment effect.

Finally, the sampling included 10 % of all primary schools in Lower Austria in both rural and urban areas. The population of Lower Austria is similar to the population of central Europe, so the results of this study may apply to many Western countries. However, differences between school systems in different countries might affect the generalisability of our findings.

## Conclusions

Considering the strong study design, we conclude that despite small statistically significant differences in motor skills, the intervention (Classes in Motion) does not have a relevant effect on the expected outcomes on individual level. The observed significant but small effects in motor skills could have the potential to produce public health benefits at the population level. Hovewer these findings reflect a need to revise and improve the intervention “Classes in Motion”.

## Abbreviations

BMI, body mass index; FIML, Full information maximum likelihood estimation; HPS, World Health Organization’s Health Promoting Schools framework; Hrs, hours; MAR, missing at random; MVPA, moderate-to vigorous physical activity
